# Inhibition of the CD47-SIRPα axis for cancer therapy: A systematic review and meta-analysis of emerging clinical data

**DOI:** 10.3389/fimmu.2022.1027235

**Published:** 2022-11-11

**Authors:** Ji Son, Rodney Cheng-En Hsieh, Heather Y. Lin, Kate J. Krause, Ying Yuan, Amadeo B. Biter, James Welsh, Michael A. Curran, David S. Hong

**Affiliations:** ^1^ Departments of Gynecologic Oncology and Reproductive Medicine, The University of Texas MD Anderson Cancer Center, Houston, TX, United States; ^2^ Departments of Immunology, The University of Texas MD Anderson Cancer Center, Houston, TX, United States; ^3^ Department of Radiation Oncology, Chang Gung Memorial Hospital at Linkou and Chang Gung University, Taoyuan, Taiwan; ^4^ Departments of Biostatistics, The University of Texas MD Anderson Cancer Center, Houston, TX, United States; ^5^ Departments of Research Medical Library, The University of Texas MD Anderson Cancer Center, Houston, TX, United States; ^6^ Departments of Investigational Cancer Therapeutics, The University of Texas MD Anderson Cancer Center, Houston, TX, United States; ^7^ Departments of Radiation Oncology, The University of Texas MD Anderson Cancer Center, Houston, TX, United States

**Keywords:** CD47 inhibitor, SIRPα inhibitor, next generation checkpoint inhibition, checkpoint inhibitor resistance, clinical trial, novel therapeutics

## Abstract

CD47-SIRPα interaction acts as a “don’t eat me” signal and is exploited by cancer to downregulate innate and adaptive immune surveillance. There has been intense interest to develop a mechanism of blockade, and we aimed to analyze the emerging data from early clinical trials. We performed a systematic review and meta-analysis of relevant databases and conference abstracts including clinical trials using CD47 and/or SIRPα inhibitors in cancer treatment. Nonlinear mixed models were applied for comparison of response and toxicity. We retrieved 317 articles, 24 of which were eligible. These included 771 response-evaluable patients with hematologic (47.1%) and solid tumors (52.9%). Of these, 6.4% experienced complete response, 10.4% partial response, and 26.1% stable disease for a 16.7% objective response rate (ORR), 42.8% disease control rate, and 4.8-month median duration of response. ORR was significantly higher for hematologic cancers (25.3%) than solid cancers (9.1%, p=0.042). Comparing by mechanism, seven CD47 monoclonal antibodies (mAbs) and six selective SIRPα blockers were given alone or combined with checkpoint inhibitors, targeted therapy, and/or chemotherapy. In solid cancers, selective SIRPα blockade showed a higher ORR (16.2%) than anti-CD47 mAbs (2.8%, p=0.079), which was significant for combination therapies (ORR 28.3% vs 3.0%, respectively, p=0.010). Responses were seen in head and neck, colorectal, endometrial, ovarian, hepatocellular, non-small cell lung, and HER2+gastroesophageal cancers. Dose-limiting toxicity (DLT) was seen in 3.3% of patients (5.4% anti-CD47 mAbs, 1.4% selective SIRPα blockers; p=0.01). The frequency of treatment-related adverse events (TRAEs) ≥grade 3 was 18.0%, similar between the two groups (p=0.082), and mostly laboratory abnormalities. For anti-CD47 mAbs, the most common toxicities included grade 1-2 fatigue (27.2%), headache (21.0%), and anemia (20.5%). For selective SIRPα blockers, these included grade 1-2 infusion reaction (23.1%) and fatigue (15.8%). Anti-CD47 mAbs were significantly more likely than selective SIRPα blockers to cause grade 1-2 fever, chills, nausea/vomiting, headache, and anemia. In conclusion, combination therapies using selective SIRPα blockade had higher response rates in solid tumors than anti-CD47 mAb combinations. Hematologic changes were the main TRAEs, and selective SIRPα blockers seemed to have a better grade 1-2 toxicity profile. Treatment was well-tolerated with minimal DLTs.

## Introduction

Immune checkpoint inhibitors (CPIs) have shown unprecedented clinical activity and revolutionized cancer care. CPIs reinvigorate anti-tumor immune responses by disrupting co-inhibitory T cell signaling, and significant durable responses have been observed. Yet, most patients’ disease fails to respond or initially responds only to give way to progression (1). Factors contributing to the poor immunogenicity of these so-called “cold tumors” include inadequate tumor-associated antigen uptake and impaired cross-priming capability of antigen-presenting cells in the tumor microenvironment, which, in turn, fosters insufficient production of antigen-specific CD8 T cells, functional incompetence of T effectors, and unsustainable antitumor immune memory (2). In light of this limitation, there has been recent interest in therapeutic targeting of the CD47-SIRPα pathway. Inhibition of this interaction, which is heralded as a “myeloid checkpoint,” provides a unique mechanism for targeting the innate immune system.

The CD47-SIRPα axis acts as a “don't eat me” signal and is an essential component of self-tolerance in normal tissue. CD47’s interaction with SIRPα negatively regulates phagocytosis in dendritic cells and macrophages and contributes to homeostasis in T cells and natural killer cells. Both hematologic and solid cancer cells exploit this pathway by overexpressing CD47, which leads to downregulation of immune surveillance and decreased immunogenicity (3, 4). In fact, the level of CD47 overexpression has been directly correlated to poor patient survival outcomes (5). In preclinical studies, inhibition of SIRPα signaling in CD8α+ type I conventional dendritic cells has been shown to enhance sensing of phagocytosed tumor mitochondrial DNA, which triggers the cGAS/STING-mediated type I interferon response that facilitates cross-presentation of tumor antigens to CD8 T cells (6, 7). *In vivo*, disruption of the CD47-SIRPα interaction enhances antibody-dependent cellular toxicity (8), dendritic cell-mediated cross-priming of T cell response (6), and cytotoxic capacity of NK cells (9). Moreover, when mice previously treated with the SIRPα inhibitor SL-172154 were rechallenged with a second colorectal tumor on the opposite flank, 60% of the mice rejected the tumor, suggesting development of T cell immune memory (10). Given the central role of the CD47-SIRPα pathway in both innate and adaptive immunity against cancer, several agents that block this interaction have entered the clinical space, with numerous others in development.

Inhibitors targeting the CD47-SIRPα axis in clinical development can be categorized by mechanism of action. The CD47 inhibitors that have been investigated thus far are predominantly humanized monoclonal IgG4 antibodies lacking effector function which target CD47 (henceforth, anti-CD47 mAb). Recent interest has focused on specifically blocking the SIRPα interaction with CD47. CD47 is ubiquitously expressed on most cells, especially those of hematopoietic lineage. Thus, anti-CD47 mAb can trigger antibody-dependent cellular cytotoxicity (ADCC) and phagocytosis (ADCP) towards normal cells in an Fc receptor-dependent manner, posing concerns for on-target adverse events such as anemia and thrombocytopenia. In contrast, SIRPα is almost exclusively present on myeloid populations and microglia. Therefore, SIRPα blockade theoretically provides a more targeted inhibition of the CD47-SIRPα axis (11). Further, the preservation of CD47-SIRPγ interaction may benefit anti-tumor immunity (12). Such selective SIRPα blockers in clinical development include SIRPα-IgG or SIRPα-Fc fusion proteins and monoclonal antibodies targeting SIRPα.

As the first wave of clinical trials targeting the CD47-SIRPα pathway are now reporting preliminary results, we conducted a systematic review and meta-analysis to summarize and analyze the emerging clinical data.

## Methods

### Systematic review

We performed a comprehensive systematic search of Ovid MEDLINE, Ovid EMBASE, Cochrane Library, ClinicalTrials.gov, and International Clinical Trials Registry. In addition, we searched “gray literature” resources from conferences, dissertations, reports, and other relevant citations. Searches were restricted to clinical trials and English-language articles. There was no time restriction. Search structures, subject headings, and keywords were tailored to each database by a medical research librarian (KJK) specializing in systematic reviews. MeSH, Emtree, and keywords were searched to identify concepts related to CD47 and/or SIRPα inhibition in human subjects. The full search strings for all databases can be found in the **Supplementary Index (S1)**.

After the initial search, Rayyan was used to screen citations (13). Studies that passed the initial title and abstract review were retrieved for full-text review. Eligible studies included clinical trials of any phase using a CD47 and/or SIRPα inhibitor reporting standardized oncologic response and/or toxicity data. We excluded animal or *in vitro* studies, multiple reports of the same data, review articles, meta-analyses, and non-peer reviewed literature such as editorials. The PRISMA flow diagram is presented in **Supplementary Figure S2** (14). We then classified the CD47 and SIRPα inhibitors by mechanism of action: anti-CD47 mAb targeting CD47 globally, and selective SIRPα blockers targeting the CD47 interaction with SIRPα.

### Data extraction

Study characteristics were collected, including type of drug, phase of trial, treatment regimen, and criteria for tumor response and toxicity. Patient and response characteristics collected included types of cancer, number of patients by cancer type, number of prior treatments, number of patients experiencing treatment response, duration of response, and survival data if available. Treatment-related adverse events (TRAEs) were collected according to Common Terminology Criteria for Adverse Events. Dose-limiting toxicities (DLTs), and serious adverse events (SAE) were also collected.

Objective response rate (ORR) was defined as the sum of complete response (CR) rate and partial response (PR) rate, and disease control rate (DCR) was defined as the sum of ORR and the stable disease (SD) rate. Missing data were assumed to be progressive disease for conservative estimation of ORR and DCR. For toxicity calculations, missing data were omitted.

### Statistical analysis

Nonlinear mixed models were applied for comparison of ORR and DCR, with study as a random effect, in the entire cohort as well as subgroups. The subgroups were defined by tumor type (hematologic vs solid) or by regimens (monotherapy vs combinatorial). Patient-level toxicity data were compared using nonlinear mixed models in a similar fashion. Statistical analysis was calculated using SAS version 9.4 (SAS Institute, Cary NC). All p values were two-sided, with 0.05 as the cutoff for level of significance.

## Results

### Study-level characteristics

We retrieved 317 articles, of which 24 articles met our eligibility criteria. 14 reported on trials of anti-CD47 mAbs and 10 on trials of selective SIRPα blockers. All trials were phase I with the exception of three combined phase I/II trials, and published between 2019-2022. Seven anti-CD47 mAbs were studied, including AK-117, AO-176, CC-90002, Hu5F9-G4, IBI188, SRF231, and TJC4 in monotherapy or in combination with CPI (avelumab), targeted therapy (rituximab, cetuximab), or chemotherapy (azacitidine). Six selective SIRPα blockers were studied, including ALX148, BI 765063, IMM-01, SL-172154, TTI-621, and TTI-622 as monotherapy or combined with CPI (pembrolizumab, ezabenlimab, nivolumab), targeted therapy (rituximab, trastuzumab, ramucirumab), or chemotherapy (platinum-based drugs, paclitaxel, 5-fluorouracil). The selective SIRPα blockers’ mechanism of action included SIRPα-Fc fusion protein with or without inactivated Fc region, SIRPα-Fc-CD40L fusion protein, SIRPα-IgG fusion protein, and monoclonal antibody targeting SIRPα. Cancer types, lines of treatment, sample size, and study-level response in each trial is listed in **Table 1**.

**Table 1:** Eligible studies including clinical trials for CD47-SIRPα inhibitors and their study-level efficacy.

Clinical trial identifier	Phase	Drug name	Mechanism (15)	Combination therapy	Cancer types included^a^	Cancer types w/response	N*	ORR
NCT04349969 (16)	I	AK117	anti-CD47 mAb	–	Solid tumor	NOS	0	–
NCT03834948 (17)	I/II	AO-176	anti-CD47 mAb	–	Solid tumor	Endometrial	27	3.7
NCT02641002 (18)	I	CC-90002	anti-CD47 mAb	–	AML	–	24	0
NCT02367196 (19)	I	CC-90002	anti-CD47 mAb	Rituximab	NHL	NHL	24	12.5
NCT03248479 (20)	I	Hu5F9-G4	anti-CD47 mAb	–	AML	–	15	0
NCT02216409 (21)	I	Hu5F9-G4	anti-CD47 mAb	–	Solid tumor	Ovarian	52	3.8
NCT03013218 (22)	I	Hu5F9-G4	anti-CD47 mAb	Azacitidine	Treatment-naíve AML	AML	34	64.7
NCT02953509 (23)	I	Hu5F9-G4	anti-CD47 mAb	Rituximab	NHL	NHL	22	50.0
NCT03558139 (24)	I	Hu5F9-G4	anti-CD47 mAb	Avelumab	Solid tumor, ovarian	Adenocarcinoma of finger	31	3.2
NCT02953782 (25)	I/II	Hu5F9-G4	anti-CD47 mAb	Cetuximab	Colorectal	Colorectal	70	2.9
NCT03763149 (26)	I	IBI188	anti-CD47 mAb	–	Solid tumor, lymphoma	NOS	0	–
NCT03512340 (27)	I	SRF231	anti-CD47 mAb	–	Solid tumor	–	37	0
NCT04202003 (28)	I/II	TJC4	anti-CD47 mAb	–	AML	AML	5	20
NCT03934814 (29)	I	TJC4	anti-CD47 mAb	–	Solid tumor	NOS	0	–
NCT03013218 (30–32)	I	ALX148	SIRPα-inactive Fc fusion protein	Rituximab	NHL	NHL	33	48.5
				–	Solid tumor	–	26	0
				Pembrolizumab	ENT, NSCLC	ENT, NSCLC	40	12.5
				Trastuzumab	HER2+ G/GE	HER2+ G/GE	19	21.1
				Pembrolizumab, 5FU, platinum	Treatment-naïve ENT	Treatment-naïve ENT	13	38.5
				Trastuzumab, ramucirumab, paclitaxel	HER2+ G/GE	HER2+ G/GE,	18	72.2
NCT 03990233 (33–35)	I	BI 765063	SIRPα mAb	–	Solid tumor	HCC	47	2.1
				Ezabenlimab	SIRPα selected GI, Gyn, NSCLC, melanoma	Colorectal, endometrial	16	18.8
ChiCTR1900024904 (36)	I	IMM-01	SIRPα-IgG1 fusion protein	–	Lymphoma	NHL, HL	12	16.7
NCT04406623 (37)	I	SL-172154	SIRPα-Fc-CD40L fusion protein	–	Ovarian	–	12	0
NCT03530683 (38)	I	TTI-622	SIRPα-IgG4 Fc fusion protein	–	Lymphoma	NHL	25	20
NCT02890368 (39)	I	TTI-621 intratumor	SIRPα-IgG1 Fc fusion protein	–	Mycosis fungoides, Sezary syndrome	Mycosis fungoides, Sezary syndrome	29	34.5
NCT02663518 (40)	I	TTI-621	SIRPα-IgG1 Fc fusion protein	–	Hematologic	NHL, HL, AML	101	11.9
				Rituximab or nivolumab	NHL or HL	NHL, HL	39	25.6

a Recurrent/metastatic unless otherwise specified.

*Evaluable patients for efficacy.

AML, acute myeloid leukemia; ENT, head and neck; G/GE, gastric and gastroesophageal; GI, gastrointestinal; Gyn, gynecologic; HCC, hepatocellular carcinoma; HL, Hodgkin lymphoma; mAb, monoclonal antibody; NHL, non-Hodgkin lymphoma; NSCLC, non-small cell lung cancer; NOS, not otherwise specified.

### Pooled patient-level efficacy

**Table 2** shows pooled patient-level efficacy data. Eligible studies included a total of 771 response-evaluable patients with hematologic (47.1%) and solid tumors (52.9%). Of these, 6.4% experienced CR, 10.4% experienced PR, and 26.1% experienced SD, for a 16.7% ORR, and 42.8% DCR. The median duration of response was 4.8 months. In all, response in hematologic cancers was higher than in solid cancers (ORR 25.3% vs 9.1%, p=0.042; DCR 56.7% vs 30.4%, p=0.097). Anti-CD47 mAbs and selective SIRPα blockers had similar efficacy in hematologic cancers, with ORRs of 29.8% and 23.0%, respectively (p=0.48, DCR p=0.69). CR or PR was seen in myelodysplastic syndrome, acute myeloid leukemia (AML), non-Hodgkin lymphoma not otherwise specified, mycosis fungoides, Sezary syndrome, diffuse large B cell lymphoma, and peripheral and cutaneous lymphoma. For solid cancers, there was a nonsignificant but notable difference in ORR between patients treated with selective SIRPα blockers (16.2%) versus anti-CD47 mAbs (2.8%, p=0.079); this was also reflected in DCR (41.9% vs 20.3%, respectively; p=0.058). Cancer types that showed CR or PR in anti-CD47 mAb studies included colorectal (n=2), ovarian (n=2), and endometrial (n=1) cancers and adenocarcinoma of the finger (n=1); in selective SIRPα blocker studies, these included HER2+ gastroesophageal (n=13), head and neck (n=9), colorectal (n=1), endometrial (n=1), non-small cell lung (n=1), and hepatocellular (n=1) cancers.

**Table 2:** Pooled patient-level data on the efficacy of CD47-SIRPα inhibitors in clinical trials.

	Total	Anti-CD47 mAbs	Selective SIRPα blockers	p value
**Total (n)**	771	341	430	
ORR (%)	16.7	12.6	20.0	0.11
DCR (%)	42.8	32.6	50.9	0.19
**Hematologic cancer (n)**	363	124	239	
ORR (%)	25.3	29.8	23.0	0.48
DCR (%)	56.7	54.0	58.2	0.69
**Solid cancer (n)**	408	217	191	
ORR (%)	9.1	2.8	16.2	0.079
DCR (%)	30.4	20.3	41.9	0.058
**Monotherapy in solid cancer (n)**	201	116	85	
ORR (%)	2.0	2.6	1.2	0.51
DCR (%)	21.4	11.2	35.3	0.081
**Combination in solid cancer (n)**	207	101	106	
ORR (%)	15.9	3.0	28.3	0.01*
DCR (%)	39.1	30.7	47.2	0.28

*p<0.05.

mAb, monoclonal antibody; ORR, objective response rate; DCR, disease control rate.

### Combination therapy in solid cancers

To analyze the contribution of combination regimens on the success of CD47-SIRPα blockade in solid tumors, studies using monotherapy were separated from combination therapies. The ORRs were similar in anti-CD47 mAb (2.6%) and selective SIRPα blockers (1.2%) used as a single agent in solid cancer (p=0.51); DCRs also did not differ significantly (11.2% vs 35.3%, respectively; p=0.081). In contrast, combination therapy with selective SIRPα blockers in solid cancers showed a significantly higher ORR of 28.3% compared to 3.0% in anti-CD47 mAb combinations (p=0.010). Selective SIRPα combination therapy with CPIs (ezabenlimab, pembrolizumab) yielded study-level ORR of 18.8% to 20.0% in patients with recurrent colorectal, endometrial, and head and neck cancers (**Table 3**) (30, 33, 34). Similar ORR of 21.1% was seen in the study combining a selective SIRPα blocker with trastuzumab in recurrent HER2+ gastroesophageal cancer patients (30). The highest ORR was seen in studies also combining cytotoxic chemotherapy: 38.5% in combination with pembrolizumab, 5-fluorouracil, and platinum drugs in treatment-naïve head and neck cancers (DCR 84.6%), and 72.2% in combination with trastuzumab, ramucirumab, and paclitaxel in recurrent HER2+ gastroesophageal cancers (DCR 88.9%) (31). Combination treatment with pembrolizumab in non-small cell lung cancer was a notable exception to the improved ORR of selective SIRPα combination therapy (ORR 5.0%, DCR 40.0%) (30).

**Table 3:** Study-level data on the efficacy of selective SIRPα blocker combination therapy in solid cancers.

Drug	Cancer type^a^	N	ORR, %	DCR, %
BI 765063, ezabenlimab (33, 34)	Solid tumors^b^	16	18.8	25.0
ALX148, pembrolizumab (30)	NSCLC	20	5.0	40.0
ALX148, pembrolizumab (30)	Head and neck, recurrent	20	20.0	30.0
ALX148, trastuzumab (30)	Gastroesophageal, HER2+	19	21.1	26.3
ALX148, pembrolizumab, 5FU, platinum (31)	Head and neck, treatment-naïve	13	38.5	84.6
ALX148, trastuzumab, ramucirumab, paclitaxel (31)	Gastroesophageal, HER2+	18	72.2	88.9

a Recurrent/metastatic unless otherwise specified.

b Partial response in colorectal and endometrial cancer patients for 12 weeks (ongoing).

ORR, objective response rate; DCR, disease control rate; NR, not reported; GI, gastrointestinal; Gyn, gynecologic; NSCLC, non-small cell lung cancer; 5FU, 5-fluorouracil.

### Pooled patient-level toxicity

**Table 4** shows pooled patient-level toxicity data reported in the eligible studies. In total, there were 942 patients for whom toxicity could be evaluated. DLTs were seen in 3.3% of patients, with a significant difference between anti-CD47 mAbs and selective SIRPα blockers (5.4% vs 1.4%, respectively; p=0.01). The frequencies of grade 3 or higher TRAEs were not different (19.2% for anti-CD47 mAbs vs 17.0% for selective SIRPα blockers, p=0.082). As expected from the physiology of CD47 expression, hematologic changes were the most common grade 3 or higher TRAEs; neutropenia (6.5%) and thrombocytopenia (8.3%) were the most common in anti-CD47 mAbs and selective SIRPα blockers, respectively. Other grade 3 or higher TRAEs reported in more than 1 patient included infusion-related reaction (IRR), increased bilirubin, increased amylase/lipase, and hypotension (all <2%). For anti-CD47 mAbs, the most common toxicities included grade 1-2 fatigue (27.2%), headache (21.0%), anemia (20.5%), and IRR (17.6%). For selective SIRPα blockers, these included grade 1-2 IRR (23.1%) and fatigue (15.8%). For grade 1-2 toxicities between the groups, anti-CD47 mAbs were significantly more likely than selective SIRPα blockers to cause fever (12.9 vs 5.7%, p=0.042), chills (13.2 vs 8.1%, p=0.002), nausea/vomiting (14.5 vs 5.9%, p=0.005), headache (21.0 vs 4.9%, p=0.002), and anemia (20.5 vs 4.0%, p=0.0002). Other serious adverse events (SAE) included febrile neutropenia (1.4%), infection (1.1%), pneumonia (0.6%), pancreatitis (0.4%), failure to thrive (0.4%), and, in 1 patient each (0.1%) of hemolytic anemia, lactic acidosis, dyspnea, pulmonary embolism, peripheral neuropathy, and death of unknown cause.

**Table 4:** Pooled patient-level toxicity data of CD47-SIRPα inhibitors in clinical trials.

	Total patients (n=942, %)	Anti-CD47 mAbs (n=448, %)	Selective SIRPɑ blockers (n=494, %)	p value
DLT	31 (3.3)	24 (5.4)	7 (1.4)	0.01*
SAE	63 (6.7)	34 (7.6)	29 (5.9)	0.11
TRAE grade 3	170 (18.0)	86 (19.2)	84 (17.0)	0.082
**TRAE Grade 3-4**				
Thrombocytopenia	57 (6.1)	16 (3.6)	41 (8.3)	0.62
Neutropenia	52 (5.5)	29 (6.5)	23 (4.7)	0.98
Anemia	43 (4.6)	26 (5.8)	17 (3.4)	0.24
IRR	11 (1.2)	6 (1.3)	5 (1)	0.41
Bilirubin inc	4 (0.4)	4 (0.9)	0 (0)	
Amylase/lipase inc	3 (0.3)	1 (0.2)	2 (0.4)	0.89
Fatigue	2 (0.2)	1 (0.2)	1 (0.2)	0.58
Hypotension	2 (0.2)	0 (0)	2 (0.4)	
Chills	1 (0.1)	1 (0.2)	0 (0)	
Diarrhea	1 (0.1)	0 (0)	1 (0.2)	
Fever	1 (0.1)	1 (0.2)	0 (0)	
Nausea/vomiting	1 (0.1)	0 (0)	1 (0.2)	
Headache	1 (0.1)	1 (0.2)	0 (0)	
LFT inc	1 (0.1)	0 (0)	1 (0.2)	
Rash	1 (0.1)	0 (0)	1 (0.2)	
Electrolyte change	0 (0)	0 (0)	0 (0)	
**TRAE Grade 1-2**				
Fatigue	200 (21.2)	122 (27.2)	78 (15.8)	0.11
IRR	193 (20.5)	79 (17.6)	114 (23.1)	0.32
Headache	118 (12.5)	94 (21.0)	24 (4.9)	0.002*
Anemia	112 (11.9)	92 (20.5)	20 (4.0)	0.0002*
Chills	99 (10.5)	59 (13.2)	40 (8.1)	0.002*
Nausea/vomiting	94 (10)	65 (14.5)	29 (5.9)	0.005*
Fever	86 (9.1)	58 (12.9)	28 (5.7)	0.042*
Thrombocytopenia	60 (6.4)	34 (7.6)	26 (5.3)	0.065
Diarrhea	56 (5.9)	34 (7.6)	22 (4.5)	0.21
Rash	46 (4.9)	29 (6.5)	17 (3.4)	0.24
Bilirubin inc	22 (2.3)	22 (4.9)	0 (0)	
Neutropenia	22 (2.3)	14 (3.1)	8 (1.6)	0.15
LFT inc	19 (2)	0 (0)	19 (3.8)	
Arthralgia	18 (1.9)	8 (1.8)	10 (2)	0.65
Pruritus	17 (1.8)	0 (0)	17 (3.4)	
Amylase/lipase inc	11 (1.2)	10 (2.2)	1 (0.2)	0.99
Anorexia	9 (1)	0 (0)	9 (1.8)	
Hypotension	6 (0.6)	0 (0)	6 (1.2)	
Electrolyte change	2 (0.2)	0 (0)	2 (0.4)	
Pneumonitis	1 (0.1)	0 (0)	1 (0.2)	

*p<0.05.

DLT, dose-limiting toxicity; inc, increased; IRR, infusion-related reaction; LFT, liver function test; mAb, monoclonal antibody; SAE, serious adverse event; TRAE, treatment-related adverse events.

## Discussion

The CD47-SIRPα pathway is an emerging target for myeloid checkpoint inhibition. In our analysis, greater response was seen in hematologic cancers, with similar rates for both anti-CD47 mAbs and selective SIRPα blockers. In solid cancers, selective SIRPα blockers yielded higher rates of response than did anti-CD47 mAbs, in large part due to the success of combination therapies. Selective SIRPα blockers seemed to have a better grade 1-2 toxicity profile, but treatment was well tolerated in all groups.

Because CD47 is ubiquitously expressed, particularly in hematopoietic cells (41, 42), consideration of on-target side effects such as anemia and thrombocytopenia have been central to inhibitor development. To mitigate on-target side effects, the majority of anti-CD47 mAb (including AK117, CC-90002, Hu5F9-G4, IBI188, SRF231, and TJC4) are composed of humanized IgG4 which has lower binding affinity for the activating FcγR (43). Nonetheless, earlier studies, including a phase I trial of CC-90002 in AML patients, were closed in part due to concerns about toxicity and the development of anti-drug antibodies (18). In patients receiving escalating doses of Hu5F9-G4, decline in hemoglobin was observed with a median change of -1.0 g/dL, correlating with increased transfusion requirement. In addition, red blood cell agglutination was seen with development of new antibodies and invalid ABO blood grouping (44). However, hemagglutination was not associated with significant clinical toxicity (21). The most advanced anti-CD47 mAb in development is Hu5F9-G4 (magrolimab), which the US Food and Drug Administration granted fast track designation for AML and myelodysplastic syndrome. This was based on results from a phase Ib trial of the drug combined with azacitidine showing an ORR of 64% in AML and 100% in myelodysplastic syndrome (22). Phase III confirmatory trials are in progress. Therapy with Hu5F9-G4 uses a unique priming and maintenance dosing strategy, which mitigates anemia by utilizing the timing of compensatory reticulocytosis since younger red blood cells display lower pro-phagocytic molecules and resistance to phagocytic clearance (11). Blood transfusions were rarely necessary in the population of solid tumor patients, whose rate of grade 3 or higher anemia was 9.7% (21). In early reports of IgG4 isotypes of anti-CD47 mAb, treatment-related anemia for AK117 (16), TJC4 (28), and IBI188 (26) were 40%, 40%, and 15% for grade 1-2 and 0%, 0%, and 5% for grade 3, respectively. For humanized IgG2 anti-CD47 mAb, AO-176 preferentially binds integrin-β1 expressed tumor cells with a lower affinity to red blood cells, and treatment-related anemia of any grade was 22% (17).

Efforts to minimize on-target toxicity led to the development of selective SIRPα blockers. Several different mechanisms of inhibition have been exploited against SIRPα interaction, including monoclonal antibodies and SIRPα-Fc fusion proteins. For instance, ALX148 contains an inactivated Fc domain which prevents Fc-dependent phagocytosis towards the opsonized normal cells while still ensuring an antibody-like pharmacokinetics with a longer half-life (45). TTI-621 is composed of the N-terminal portion of SIRPα, which demonstrates lower binding affinity to human erythrocytes minimizing the risk of anemia (46). In published trial results, the rates of DLT and grade 1-2 anemia were significantly lower for selective SIRPα blockers. However, because of inconsistent reporting of grade 1-2 TRAE, definitive conclusions are limited. Overall, the rate of high grade hematologic toxicity was less than 10% for both groups.

Consistent with preclinical studies, monotherapy with CD47 or SIRPα inhibitors demonstrated unsatisfactory therapeutic efficacy in patients with solid tumors, with ORRs less than 5% (17, 21, 27, 30, 35, 37). Combination therapy significantly improved response. Because CD8 T cells are upregulated in response to CD47-SIRPα blockade, PD-L1/PD-1 inhibitors have been proposed to synergistically augment both innate and adaptive antitumor response (47). SIRPα combination therapy with CPI (ezabenlimab, pembrolizumab) yielded a study-level ORR of 18.8% to 20.0% in colorectal, endometrial, and head and neck cancer patients. The three partial responses shown in a study using BI 765063 are noteworthy in that all patients were microsatellite stable (33, 34). Given the limited efficacy of CPI alone for microsatellite stable cancers with ORR ranging from 0-7% (48–50), these promising findings suggest that dual blockade of SIRPα and PD-1 may augment phagocytosis and play a role in overcoming CPI resistance.

Furthermore, physiologic initiation of phagocytosis requires expression of pro-phagocytic molecules, such as damage-associated molecular patterns, on tumor cells in addition to the absence of inhibitory signals. Emerging evidence indicates that αCD47 or αSIRPα monotherapy exerts limited therapeutic efficacy due to lack of endogenous pro-phagocytic “eat me” triggers on tumor cells (11, 51). Hence, combining a cytotoxic therapy to induce the display of activating signals has shown to magnify the therapeutic advantages of phagocytosis checkpoint blockade against poorly immunogenic tumors. Targeted therapies such as cetuximab or trastuzumab have shown success by inducing both ADCC and ADCP through Fc receptor interaction (5, 52), which translated clinically (**Table 3**). Moreover, several successful combinations with chemotherapy have been presented. These include the combination of ALX148 with trastuzumab and chemotherapy in HER2+ gastroesophageal cancer, which has shown a remarkable ORR of up to 72% (53, 54). While difficult to generalize due to patient selection and lack of a comparison arm, this favorable outcome relative to monotherapy implies that CD47-SIRPα inhibition is more effective when combined with cytotoxic therapy.

Intriguingly, various preclinical studies suggested that selective SIRPα blockade is more efficacious than CD47 inhibition in solid tumors (51, 55). The lower therapeutic effect of anti-CD47 mAbs may result from antibody sequestration by the healthy tissue “sink”, which thereby limits the blockade efficiency against the tumor cells. More importantly, CD47 also engages SIRPγ, which is pivotal for T cell endothelial transmigration, cell-cell adhesion, and costimulation between T cells and dendritic cells (56, 57). Global inhibition of CD47 compromises SIRPγ-mediated T cell activation, proliferation, and migration, resulting in a dampened antitumor T cell response. In contrast, selective SIRPα blockade may preserve these essential T cell functions, allowing higher T cell infiltration and pro-inflammatory cytokine production (12). The role of binding affinity as it relates to clinical efficacy, especially in the more newly developed fusion proteins and bispecific antibodies, is complex (52, 58), and will become apparent as pharmacokinetic and pharmacodynamic data from the ongoing trials become available. In all, due to the relatively limited array of combination therapies studied in anti-CD47 mAbs for solid cancers, differing patient enrollment criteria, and heterogeneous drug designs, conclusions regarding the inherent superiority of selective SIRPα blockers in this setting cannot be drawn.

Finally, patient selection seems to be an important component of response and may therefore direct future research. In general, most cancer types with CD47 alteration/amplification, such as ovarian, esophageal, endometrial, and head and neck cancers (59), seemed to respond to CD47-SIRPα blockade. However, non-small cell lung cancer was a notable exception with a 5.0% ORR in combination therapy (30). Results from ongoing pharmacodynamic studies may shed light on predictive biomarkers. For example, patient selection based on expression of SIRPα V1 allele was a novel approach (33). Furthermore, future drug design should focus on the specificity of SIRPα inhibition so as to minimize on-target side effects (52). While not yet observed, theoretical toxicity due to the minor expression of SIRPα in the central and peripheral nervous system should be considered (46). Scientifically rational combination therapies to further augment the activating “eat me” signal should be explored.

Our study has some limitations. The chief limitation is that the protocols of the included studies varied considerably. This was necessary because published data in their final form are scarce for this topic. Similarly, our inclusion of gray literature, which enabled us to include the most recent data, also led to missing variables and incomplete protocol information. In particular, there was a paucity of patient-level toxicity data. Because the studies of anti-CD47 mAbs tend to predate those of selective SIRPα blockers, the former had more complete toxicity data, which may have overrepresented this effect in our analysis. We attempted to account for this by omitting missing toxicity data in statistical comparisons. In addition, the array of combination therapies seemed relatively limited in anti-CD47 mAbs, particularly combinations with cytotoxic therapy. Along this vein, the efficacy comparison of combination therapy was largely driven by one selective SIRPα blocker, ALX148. While data presented herein was statistically significant and thought-provoking, our conclusion should be considered merely hypothesis-generating. Survival and duration of response were often not available because the studies are ongoing. Similarly, pharmacokinetic and pharmacodynamic data from the source studies are much anticipated. Despite these limitations, this represents the first comprehensive review and analysis of patient-level data on CD47-SIRPα inhibitors in clinical trials.

CD47-SIRPα inhibition shows promise in cancer therapy; selective SIRPα blockade in combination with cytotoxic therapies in particular seem to maximize anti-tumoral benefit. While there was no difference in higher grade TRAEs, selective SIRPα blockers appear to have a milder grade 1-2 toxicity profile and lower DLT thus far. Treatment was well tolerated for both groups. Many clinical trials - including those in **Table 1** and others that have yet to report data, such as TQB2928, ZL-1201, STI-6643, IMC-002 among many others - are ongoing.

## Data availability statement

The original contributions presented in the study are included in the article/**Supplementary Material**. Further inquiries can be directed to the corresponding author.

## Author contributions

JS performed the study design, data collection, formal data analysis, and manuscript writing. RC-EH performed manuscript writing and editing and formal data analysis. HL performed the formal statistical analysis and manuscript editing. KK is the research librarian who performed the initial systematic search of the relevant databases. AB, YY, and JW performed manuscript review and editing. MC and DH are the senior authors who contributed conceptualization, study design, formal data analysis, and manuscript editing. All authors contributed to the article and approved the submitted version.

## Funding

The authors declare no funding directly related to this study. We acknowledge support through the MD Anderson Cancer Center Support Grant from the National Cancer Institute of the National Institutes of Health (NIH/NCI P30 CA016672, CA217685), the T32 training grant CA101642 (JS), Cancer Prevention and Research Institute of Texas (CPRIT) Research Training Grant RP170067 (RCH).

## Acknowledgments

We thank Bryan Tutt in the Research Medical Library at The University of Texas MD Anderson Cancer Center for editing this article.

## Conflict of interest

The authors declare no conflict of interest directly relating to this study. Possible competing interests outside of this work include: JS received institutional research or grant funding from NIH/NCI P30CA016672, CA217685 and the T32 training grant CA101642. RC-EH formal data analysis received institutional research or grant funding from Cancer Prevention and Research Institute of Texas CPRIT research training grant RP170067. YY has served as a statistical consultant to AbbVie, Amgen, Bexion Pharmaceuticals, BeyondSpring Pharmaceuticals, Boehringer Ingelheim Pharmaceuticals, Bristol Myers Squibb, Century Therapeutics, Enliven Therapeutics, NGM Biopharmaceuticals, Repare Therapeutics, Servier Pharmaceuticals, Starpax Pharmaceuticals, and Vertex Pharmaceuticals. JW has received institutional research or grant funding from GlaxoSmithKline, Bristol Meyers Squibb, Merck, Nanobiotix, RefleXion, Alkermes, Artidis, Mavu Pharma, Takeda, Varian, Checkmate Pharmaceuticals, HotSpot Therapeutics, Inc., Gilead and Kiromic. JW serves/served on the scientific advisory board for Legion Healthcare Partners, RefleXion Medical, MolecularMatch, Merck, AstraZeneca, Aileron Therapeutics, OncoResponse, Checkmate Pharmaceuticals, Mavu Pharma, Alpine Immune Sciences, Ventana Medical Systems, Nanobiotix, China Medical Tribune, GI Innovation, Genentech and Nanorobotix. JW serves as consultant for Lifescience Dynamics Limited. JW has/had Speaking Engagements for Ventana Medical Systems, US Oncology, Alkermes, Boehringer Ingelheim, Accuray and RSS. JW holds/held stock or ownership in Alpine Immune Sciences, Checkmate Pharmaceuticals, Healios, Mavu Pharma, Legion Healthcare Partners, MolecularMatch, Nanorobotix, OncoResponse, and RefleXion. JW has accepted honoraria in the form of travel costs from Nanobiotix, RefleXion, Varian, Shandong University, The Korea Society of Radiology, Aileron Therapeutics and Ventana. JW has the following patents; MP470 amuvatinib, MRX34 regulation of PDL1, XRT technique to overcome immune resistance. MD Anderson Cancer Center has a trademark for RadScopal™. MC has received institutional research or grant funding from ImmunoGenesis, Inc., ImmunoMet, Inc.; received consulting fees from ImmunoGenesis, Inc., Alligator Bioscience, Inc., from ImmunOS, Inc., ImmunoMet, Inc., Oncoresponse, Inc., from Pieris, Inc., from Nurix, Inc., Aptevo, Inc., Servier, Inc., Kineta, Inc., Salarius, Inc., Xencor, Inc., Agenus, Inc., Mereo, Inc., Amunix, Inc., Adagene, Inc. MC has a patent Methods and Composition for Localized Secretion of Anti-CTLA-4 Antibodies with royalties paid to multiple licensees, a patent Dual specificity antibodies which bind both PD-L1 and PD-L2 and prevent their binding to PD-1 with royalties paid to ImmunoGenesis, Inc., and a patent Cyclic Dinucleotides as Agonists of Stimulator of Interferon Gene Dependent Signaling licensed to ImmunoGenesis, Inc. DH has received institutional research or grant funding from AbbVie, Adaptimmune, Adlai-Nortye, Amgen, Astra-Zeneca, Bayer, Bristol-Myers Squibb, Daiichi-Sankyo, Deciphera, Eisai, Eli Lilly, Endeavor, Erasca, F. Hoffmann-LaRoche, Fate Therapeutics, Genentech, Genmab, Ignyta, Infinity, Kite, Kyowa Kirin, LOXO, Merck, Medimmune, Mirati, Mologen, Navier, NCI-CTEP, Novartis, Numab, Pfizer, Pyramid Bio, SeaGen, Takeda, TCR2, Teckro, Turning Point Therapeutics, and VM Oncology; held a consulting, speaker, or advisory role for Abbvie, Adaptimmune, Alpha Insights, Acuta, Alkermes, Amgen, Aumbiosciences, Axiom, Baxter, Bayer, Boxer Capital, BridgeBio, COR2ed, COG, Cowen, Ecor1, Gennao Bio, Genentech, Gilead, GLG, Group H, Guidepoint, HCW Precision, Immunogen, Infinity, Janssen, Liberium, MedaCorp, Medscape, Numab, Oncologia Brasil, Pfizer, Pharma Intelligence, POET Congress, Prime Oncology, RAIN, SeaGen, ST Cube, Takeda, Tavistock, Trieza Therapeutics, Turning Point Therapeutics, WebMD, YingLing Pharma, and Ziopharm; travel, accommodation, or expense support from AACR, ASCO, Bayer, Genmab, Gilead, SITC, and Telperian; and has other ownership interests in Molecular Match advisor, OncoResponse founder, advisor, and Telperian founder, advisor.

The remaining authors declare that the research was conducted in the absence of any commercial or financial relationships that could be construed as a potential conflict of interest.

## Publisher’s note

All claims expressed in this article are solely those of the authors and do not necessarily represent those of their affiliated organizations, or those of the publisher, the editors and the reviewers. Any product that may be evaluated in this article, or claim that may be made by its manufacturer, is not guaranteed or endorsed by the publisher.
