# Supplementary material for: Inhibition of the CD47-SIRPα axis for cancer therapy: A systematic review and meta-analysis of emerging clinical data
Source: Front Immunol. 2022 Nov 11;13:1027235. doi: 10.3389/fimmu.2022.1027235 (PMC9691650; doi:10.3389/fimmu.2022.1027235)
Supplement: Supplementary file 1 [file DataSheet_1.docx]

Supplementary Material

**Supplementary index S1.** Full search strings for all databases

**Ovid MEDLINE**

1. (SIRPa or "SIRP a" or "SIRP-1alpha" or "signal-regulator* protein alpha" or "signal-regulator* protein a" or SIRPalpha1 or "SIRP alpha1" or "SIRPalpha2" or "SIRP alpha2" or "SHPS1" or "SHPS-1" or APX701 or "CC-95251" or "BI 765063" or "BI 754091" or PTPNS1 or "Protein tyrosine phosphatase nonreceptor type substrate 1" or "myd-1" or myd1 or CD172* or "cd 172*" or "macrophage fusion receptor*").ti,ab. or ((cd47 or "cd 47") adj (inhibitor* or blockers* or antibod* or blockade* or blocking)).ti,ab. or (cd47 or "cd 47").ti.
2. randomized controlled trial.pt.
3. controlled clinical trial.pt.
4. randomized.ab.
5. placebo.ab.
6. clinical trials as topic.sh.
7. randomly.ab.
8. trial.ti.
9. exp RANDOMIZED CONTROLLED TRIAL/
10. exp RANDOMIZED CONTROLLED TRIALS AS TOPIC/
11. CROSS-OVER STUDIES/
12. CLINICAL TRIAL, PHASE I/
13. (phase adj (1* or I or Ib or "I/II")).ti.
14. CLINICAL TRIAL, PHASE II/
15. ((("phase 2*" or "phase II*") not "phase III*") and (placebo or random* or blind*)).ti,ab,kf.
16. CLINICAL TRIAL, PHASE III/
17. CLINICAL TRIALS, PHASE III AS TOPIC/
18. ("phase 3*" or "phase III*").ti,ab,kf.
19. CLINICAL TRIAL, PHASE IV/
20. (("phase 4*" or "phase IV*") and (placebo or random* or blind*)).ti,ab,kf.
21. (placebo or randomi?ed or randomly).ti,ab. not ("systematic review" or "meta-analysis").ti,ab,kf.
22. (random* adj3 (allocat* or assign* or blind* or control* or divided or picked or placebo* or select* or study or trial)).ti,ab.
23. ((crossover or cross-over or equivalence or noninferiority or non-inferiority or pragmatic or superiority) adj2 (study or trial)).ti,ab.
24. ((singl* or doubl* or tripl*) adj (blind* or mask*)).ti,ab,kf.
25. CONTROLLED CLINICAL TRIAL/
26. CONTROLLED CLINICAL TRIALS AS TOPIC/
27. (ANZCTR or ChiCTR or clinicaltrials or CRiS or CTRI or DRKS or EUDRACT or ICTRP or IRCT or ISRCTN or JPRN or mRCT or NTR or PACTR or ReBec or RPCEC or SLCTR or TCTR or UMIN or UMIN-CTR).si,ab. not ("systematic review" or "meta-analysis").ti,ab,kf.
28. (blind or blinded or placebo or random* or sham).ti,ab.
29. OR/2-28 [All possible clinical trials]
30. AND/1,29
31. exp animals/ not humans.sh.
32. 30 not 31
33. Limit 32 to english language

**PUBMED**

(("SIRPA protein, human" [Supplementary Concept] OR SIRPa[Title/Abstract] OR "SIRP a"[Title/Abstract] OR "SIRP-1alpha"[Title/Abstract] OR "signal-regulator protein alpha"[Title/Abstract] OR "signal-regulator protein a"[Title/Abstract] OR SIRPalpha1[Title/Abstract] OR "SIRP alpha1"[Title/Abstract] OR "SIRPalpha2"[Title/Abstract] OR "SIRP alpha2"[Title/Abstract] OR "SHPS1"[Title/Abstract] OR "SHPS-1"[Title/Abstract] OR APX701[Title/Abstract] OR "CC-95251" [Title/Abstract] OR "BI 765063"[Title/Abstract] OR "BI 754091"[Title/Abstract] OR PTPNS1[Title/Abstract] OR "Protein tyrosine phosphatase nonreceptor type substrate 1"[Title/Abstract] OR "myd-1"[Title/Abstract] OR myd1[Title/Abstract] OR CD172*[Title/Abstract] OR "cd 172"[Title/Abstract] OR "macrophage fusion receptor*"[Title/Abstract] OR ((cd47[Title/Abstract] or "cd 47"[Title/Abstract]) AND (inhibitor*[Title/Abstract] or blockers*[Title/Abstract] or antibod*[Title/Abstract] or blockade*[Title/Abstract] or blocking[Title/Abstract])) OR (cd47[TI] OR "cd 47"[TI])) AND ((humans[Filter]) AND (english[Filter]))) AND (("randomized controlled trial"[pt] or "controlled clinical trial"[pt] OR randomized[tiab] OR placebo[tiab] OR randomly [tiab]OR trial[tiab] OR groups[tiab] OR "drug therapy"[sh]) AND ((humans[Filter]) AND (english[Filter]))) Filters: Humans, English

**Ovid EMBASE**

1. (SIRPa or "SIRP a" or "SIRP-1alpha" or "signal-regulator* protein alpha" or "signal-regulator* protein a" or SIRPalpha1 or "SIRP alpha1" or "SIRPalpha2" or "SIRP alpha2" or "SHPS1" or "SHPS-1" or APX701 or "CC-95251" or "BI 765063" or "BI 754091" or PTPNS1 or "Protein tyrosine phosphatase nonreceptor type substrate 1" or "myd-1" or myd1 or CD172* or "cd 172*" or "macrophage fusion receptor*").ti,ab. or ((cd47 or "cd 47") adj (inhibitor* or blockers* or antibod* or blockade* or blocking)).ti,ab. or (cd47 or "cd 47").ti.
2. exp RANDOMIZED CONTROLLED TRIAL/
3. "RANDOMIZED CONTROLLED TRIAL (TOPIC)"/
4. CROSSOVER PROCEDURE/
5. DOUBLE BLIND PROCEDURE/
6. INTENTION TO TREAT ANALYSIS/
7. PARALLEL DESIGN/
8. (placebo or randomi?ed or randomly).ti,ab. not ("systematic review" or "meta-analysis").ti,ab,kw.
9. (random* adj3 (allocat* or assign* or blind* or control* or divided or picked or placebo* or select* or study or trial)).ti,ab.
10. ((crossover or cross-over or equivalence or noninferiority or non-inferiority or pragmatic or superiority) adj2 (study or trial)).ti,ab.
11. ((singl* or doubl* or tripl*) adj (blind* or mask*)).ti,ab,kw.
12. Exp CLINICAL TRIAL/
13. PHASE 1 CLINICAL TRIAL/
14. (phase adj (1* or I or Ib or "I/II")).ti.
15. PHASE 2 CLINICAL TRIAL/
16. "PHASE 2 CLINICAL TRIAL (TOPIC)"/
17. (("phase 2*" or "phase II*") not "phase III*").ti,ab,kw.
18. PHASE 3 CLINICAL TRIAL/
19. "PHASE 3 CLINICAL TRIAL (TOPIC)"/
20. ("phase 3*" or "phase III*").ti,ab,kw.
21. PHASE 4 CLINICAL TRIAL/
22. "PHASE 4 CLINICAL TRIAL (TOPIC)"/
23. ("phase 4*" or "phase IV*").ti,ab,kw.
24. INTERVENTION STUDY/
25. POSTMARKETING SURVEILLANCE/
26. CONTROLLED CLINICAL TRIAL/
27. "CONTROLLED CLINICAL TRIAL (TOPIC)"/
28. CONTROL GROUP/
29. MAJOR CLINICAL STUDY/
30. OR/2-29
31. AND/1,30
32. (exp ANIMAL/ OR exp ANIMAL MODEL/ OR exp ANIMAL EXPERIMENT/ OR EXPERIMENTAL ANIMAL/ or NON-HUMAN/) not exp HUMAN/
33. (animal? or canine or dog or dogs or murine or porcine or rabbit* or rat or rats or mouse or mice or pig or pigs or primate or primates or veterinary or zebra*).ti,ab,kw.
34. OR/32-33[animal studies]
35. 31 not 34
36. limit 35 to english language

**CLINICALTRIALS.GOV**

(SIRPa OR "SIRP a" OR "SIRP-1alpha" OR "signal-regulator* protein a*" OR SIRPalpha* OR "SIRP alpha*" OR "SHPS1" OR "SHPS-1" OR APX701 OR "CC-95251" OR "BI 765063" OR "BI 754091")

OR

(PTPNS1 OR "Protein tyrosine phosphatase nonreceptor type substrate" OR "myd-1" OR myd1 OR CD172* OR "cd 172" OR "macrophage fusion receptor*")

OR

((cd47 or "cd 47") AND (inhibitor* or blockers* or antibod* or blockade* or blocking))

**ICTRP**

SIRPa OR "SIRP a" OR "SIRP-1alpha" OR "signal-regulatory protein" OR SIRPalpha* OR "SIRP alpha*" OR "SHPS1" OR "SHPS-1" OR APX701 OR "CC-95251" OR "BI 765063" OR "BI 754091" OR PTPNS1 OR "Protein tyrosine phosphatase nonreceptor type substrate" OR "myd-1" OR myd1 OR CD172* OR "cd 172" OR "macrophage fusion receptor" OR ((cd47 or "cd 47") AND (inhibitor* or blockers* or antibod* or blockade* or blocking))

Records identified from:

- Ovid EMBASE: 217
- Medline: 110
- Cochrane: 12
- Clinic Trials.gov: 51
- ICTRP: 48

TOTAL: 438

Records removed *before screening*:

- By hand: 121
- By software: 11

Records screened
(n = 306)

Records excluded
(n = 282)

Reports sought for retrieval
(n = 24)

Reports not retrieved
(n = 0)

Reports assessed for eligibility
(n = 24)

Reports excluded
(n = 0)

Studies included in review
(n = 24)

**Identification of studies via databases and registers**

**Identification**

**Screening**

**Included**

## Supplementary Figure S2. PRISMA 2020 flow diagram of search and selection process^14^
